# Precipitating Factors and Treatment Outcomes of Hepatic Encephalopathy in Liver Cirrhosis

**DOI:** 10.7759/cureus.4363

**Published:** 2019-04-02

**Authors:** Nandu S Poudyal, Sitaram Chaudhary, Sudhamshu KC, Bidhan N Paudel, Bhupendra K Basnet, Amrendra Mandal, Paritosh Kafle, Baikuntha Chaulagai, Azad Mojahedi, Mukesh S Paudel, Barun Shrestha, Vijay Gayam

**Affiliations:** 1 Gastroenterology, National Academy of Medical Sciences, Kathmandu, NPL; 2 Hepatology, National Academy of Medical Sciences, New Delhi, IND; 3 Gastroenterology, National Academy of Medical Sciences, Kathamandu, NPL; 4 Internal Medicine, Interfaith Medical Center, Brooklyn, USA; 5 Internal Medicine, Interfaith Medical Centre, Brooklyn, USA; 6 Gastroenterology, Lumbini City Hospital, Patan, NPL; 7 Gastroenterology, Chitwan Medical College, Bharatpur, NPL

**Keywords:** hepatic encephalopathy, liver cirrhosis, precipitating factors, treatment outcome

## Abstract

Background

Hepatic encephalopathy (HE) is a common cause of hospital admission in patients with liver cirrhosis (LC). The aims of this study were to evaluate the precipitant factors and analyze the treatment outcomes of HE in LC.

Methods

All the LC patients admitted between February 2017 and January 2018 for overt HE were analyzed for precipitating factors and treatment outcomes. Treatments were compared among three treatment groups: receiving lactulose, lactulose plus L-ornithine L-aspartate (LOLA), and lactulose plus rifaximin. The primary endpoints were mortality and hospital stay. The chi-square test was used to compare the different treatment outcomes with hospital stay and mortality with significance at p<0.05.

Results

A total of 132 patients (mean age 49.2 ± 10.2 years; male/female ratio of 103:29) were studied. The most common precipitating factor of HE was infection 65 (49.2%), followed by electrolyte imbalance 54 (41%), constipation 44 (33.33%), and gastrointestinal bleeding 21 (16%) patients. At the time of admission, 29 (22%), 76 (57.5%), 21 (16%), and six (4.5%) patients had grade I, II, III, and IV HE, respectively. The difference in mortality was not statistically significant (p=0.269) in three groups but the hospital stay was shorter among patients in groups B and C than in group A alone (7.36 ± 4.58 and 7 ± 3.69, 9.64 ± 5.28 days, respectively, p=0.015).

Conclusions

Infection, especially spontaneous bacterial peritonitis, was the commonest precipitating factor of HE. The combination of lactulose either with LOLA or rifaximin is equally effective in improving HE and reducing the duration of hospital stay than lactulose alone.

## Introduction

Liver cirrhosis (LC), the final pathway for a wide variety of chronic liver diseases, is a pathologic entity defined as diffuse hepatic fibrosis with the replacement of the normal liver architecture by nodules [1]. Hepatic encephalopathy (HE) is a well-recognized clinical complication of LC and the presence and prompt identification of well-defined precipitating factors are extremely important in the diagnosis and treatment of this fatal condition.

HE develops in 50% to 70% of patients with cirrhosis, and its occurrence is a poor prognostic indicator, with projected one- and three-year survival rates of 42% and 23%, respectively, without liver transplantation [2].

Lactulose is commonly used in the treatment of HE, however, there is increasing evidence of add-on therapy with rifaximin. Additionally, there is no study conducted using L-ornithine L-aspartate (LOLA) plus lactulose with lactulose alone in our knowledge. We aimed to study the different combinations of these drugs in our population with lactulose in all the three groups as a standard of care.

## Materials and methods

A descriptive, cross-sectional, single-center study was carried out on 132 consecutive patients of HE admitted to the department of gastroenterology and liver unit at National Academy of Medical Sciences, Bir Hospital, Nepal, between February 2017 and January 2018. All patients ages ranging from 18 to 75 years with LC with HE type C, irrespective of the etiology of LC, were enrolled while LC patients with intractable HE or other identifiable brain lesions, LC with other co-morbid conditions, and death within 24 hours were excluded from the study.

LC was diagnosed on the basis of the clinical examination and biochemical and imaging diagnosis. The diagnosis of acute HE was made on the basis of a detailed history and physical examination and West Haven Criteria [3]. The clinical findings on examination, including the presence of jaundice, pallor, fever, asterixis, and ascites, were recorded. Improvement in HE was defined as the complete reversal of clinical symptoms on the basis of the West Haven Criteria.

Blood samples were sent for complete blood count, random blood sugar, liver function tests, renal function tests, serum albumin levels, hepatitis B virus (HBV), anti-hepatitis C virus (anti-HCV), human immunodeficiency virus (HIV), coagulation profile, urine analysis, and blood cultures. Findings of abdominal ultrasound, including liver and spleen size, parenchymal echogenicity, portal vein diameter, and ascites were noted. An ascitic tap was done in all patients with ascites and sent for a detailed report and culture in order to diagnose infection on ascites. Urine routine and culture were also sent in all patients, along with blood culture, in order to diagnose any infection. A chest X-ray was done in all patients on arrival at the hospital. Non-contrast computed tomography (CT) head was also done to exclude intracranial lesions, especially bleed. All other supportive investigations were done as and when needed.

After meeting the inclusion criteria, patients were divided into three groups. Group A (n= 44) patients were treated with lactulose alone as a syrup formulation, containing 40 g lactulose/60 mL, 30-60 ml, three times a day, to ensure the patient passes two to three semi-soft stools in a day. Group B (n= 44) patients were treated with lactulose and LOLA (intravenous 20 grams per day) until the patients reverted back to normal. They were then given a 5 g sachet formulation containing granules with a dose of 20 g/day until hospital stay. Group C (n=44) was treated with lactulose and rifaximin, 550 mg capsule two times a day. If the patients did not improve with the given treatment or if another drug had to be added to improve their condition, those patients were included as treatment failures. Patients were followed until they were discharged from the hospital or died during hospital stay. So the primary endpoints were mortality and hospital stay.

Patients also received other standard treatments according to their need, which included antibiotics according to the sensitivity of the culture report, electrolyte correction, and control of gastrointestinal bleed (GIB) by vasopressin analogs (octreotide or terlipressin) as initial medical management before definitive therapy with endoscopy was performed.

Data analysis and statistical methods

The data obtained from the study were analyzed using Statistical Package for Social Studies (SPSS) version 17 (IBM Corp., Armonk, NY, US). For a comparison of categorical variables, the chi-square test, independent t-test, and bivariate and multivariate analyses were used to compare the results of various parameters among the studied patients. Values were expressed as mean ± SD, a 95% confidence interval was taken, and p-values of <0.05 were considered statistically significant.

## Results

**Figure 1:**
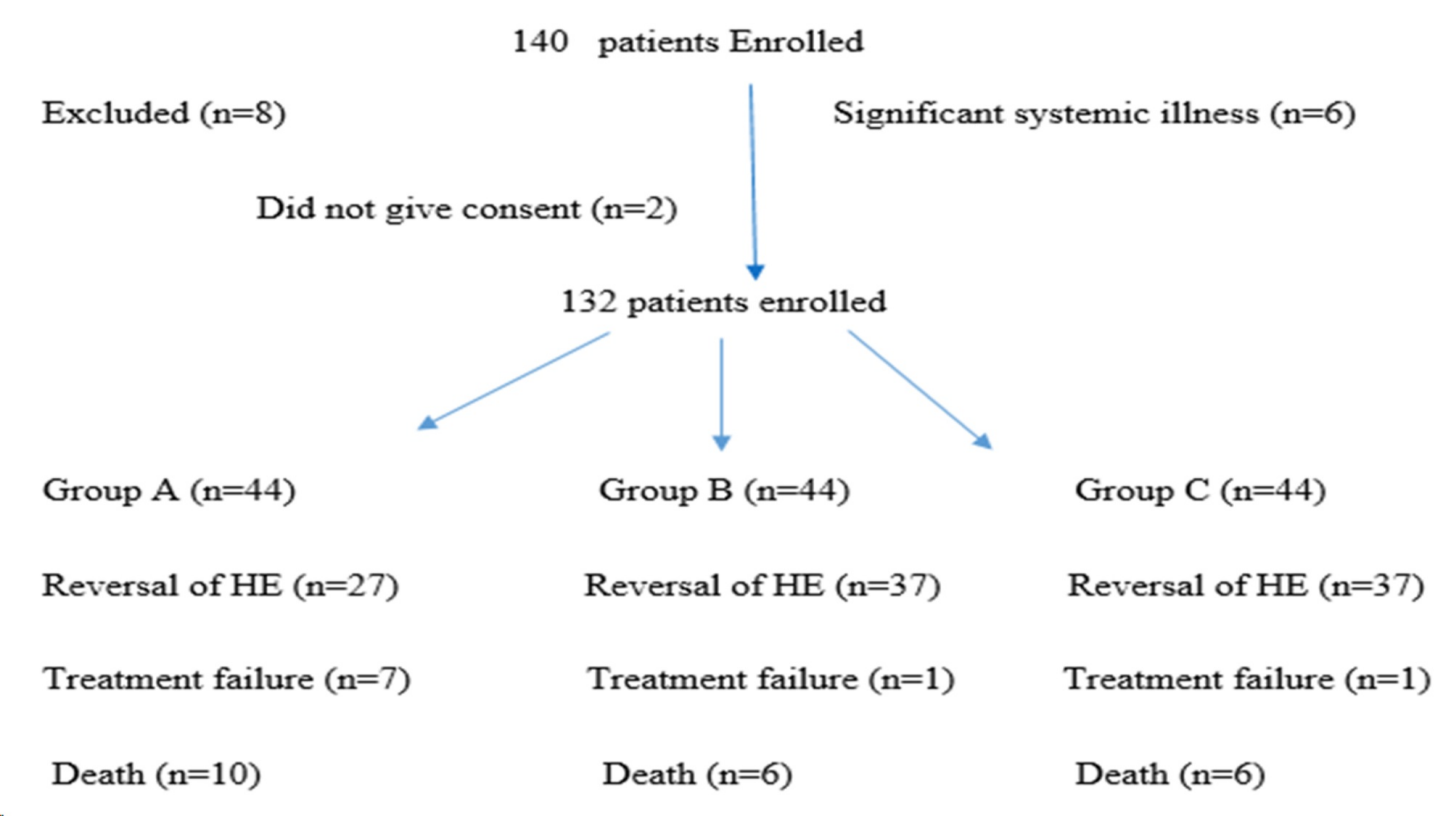
Flowchart on types of cases included in the study

A total of 140 consecutive patients with LC and HE were enrolled. Of these, eight patients were excluded because of significant systemic illness (acute respiratory distress syndrome (ARDS), septic shock) and two patients did not give consent. Finally, 132 (n=132) cases were included in the study, as shown in the flow chart in Figure 1.

The most common cause of chronic liver disease was found to be alcohol - 114 (86%), followed by hepatitis B virus - nine (7%), hepatitis C virus - six (5%), and cryptogenic - three (2%) in our study. To assess the severity of liver disease in our patients, Child-Pugh (CTP), model end-stage liver disease (MELD), MELD-Na scoring, and HE grading were applied. In this study, 38 (28.8%) patients were in CTP class B and 94 (71.2 %) were in CTP class C. The mean CTP score was 10.24 ± 1.85 and the mean MELD score was 24.5± 4.2. Twenty-nine (22%) patients had grade I, 76 (57.5%) had grade II, 21 (16%) had grade III, and six (4.5%) had grade IV HE at the time of admission (Table 1).

**Table 1:** Baseline characteristics of study patients LOLA: L-ornithine L-aspartate; HBV: hepatitis B virus; HCV: hepatitis C virus; CTP: Child-Pugh; MELD: model for end-stage liver disease; HE: hepatic encephalopathy

Parameters	Lactulose	Lactulose + Rifaximin	Lactulose + LOLA	p-value
Age (Years)	48.68±9.00	48.20±10.69	50.73±10.86	0.471
Male/Female	29:15	37:7	37:7	
Etiology				
Alcohol	39(88.6%)	36(81.8%)	38(86.3%)	
HBV	3(6.8%)	3(6.8%)	3(6.8%)	
HCV	1(2.3%)	3(6.8%)	2(4.6%)	
Others	1(2.3%)	2(4.6%)	1(2.3%)	
CTP B	9(20.5%)	18(40.9%)	11(25%)	0.084
CTP C	35(79.5%)	26(59.1%)	33(75%)	
MELD score	24.50±8.02	20.16±6.20	21.98±7.78	0.024
MELD Na	26.61±7.79	23.27±7.16	24.02±8.37	0.112
HE grade 1/2/3/4	16/21/7/0	7/28/8/1	6/27/6/5	0.02

Baseline total count, hemoglobin, platelets, blood sugar random, prothrombin time, international normalized ratio (INR), total protein, albumin, liver function test, renal function test, and serum electrolyte were comparable in the three groups (Table 2).

**Table 2:** Baseline laboratory parameters of the study patients INR: international normalized ratio; AST: aspartate aminotransferase

Parameters	Lactulose	Lactulose and LOLA	Lactulose and Rifaximin	p-value
Total Count (/cumm), mean±SD	12693.00±7903.59	11348.00±10933.47	10639.00±8206.84	0.563
Hemoglobin (gm/dL), mean±SD	9.16 ±1.85	9.48± 2.46	9.67±2.21	0.549
Platelets (/cumm), mean±SD	118890±133268.00	101970±55181.56	116670.00±53739.14	0.625
Blood sugar (mg/dl), mean±SD	99.02±30.60	116.39±47.45	117.02±105.85	0.387
Prothombin_time (secs), mean±SD	23.23±8.93	22.87±10.51	23.65±9.81	0.932
INR, mean ±SD,	1.91±0.72	1.85±0.67	1.92±0.70	0.885
Total protein (gm/dL), mean±SD	6.23±1.03	6.25±0.87	6.46±1.04	0.480
Albumin (gm/dL), mean±SD	2.45±0.52	2.53±0.50	2.44±0.54	0.670
Bilirubin total (gm/dL), mean±SD	11.30±10.43	9.13±8.86	6.51±7.26	0.046
Bilirubin direct (gm/dL), mean±SD	6.71±6.48	4.72±5.35	3.37±4.39	0.018
AST (IU/L), mean±SD	55.68±34.5	50.84±31.77	65.02±70.21	0.385
AST (IU/L), mean±SD	122.11±90.96	102.66±56.84	126.36±129.89	0.478
Alkaline phosphatase (IU/L), mean±SD	114.57±64.94	125.20±68.27	134.68±67.70	0.373
Sodium (Meq/l)	132.07±.12	129.89±9.94	132.34±10.35	0.397
Potassium (M eq/l), mean±SD	4.16±1.07	4.05±0.94	4.42±0.84	0.182
Urea (gm/dl)	67.48±50.33	47.86±27.58	69.68±41.23	0.025
Creatinine (gm/dl), mean±SD	1.63±1.25	0.97±0.34	1.62±1.10	0.002

Precipitating factors for hepatic encephalopathy 

Out of 132 patients, infection was the most common factor seen in 65 (49.2%) patients in this study. Infection in the form of spontaneous bacterial peritonitis (SBP) (18.2%) was the most common precipitant factor followed by 14.4% respiratory tract infections, 13.7% urinary tract infections, and 3% with fever of undetermined cause. Dyselectrolytemia was present in 54 (41%) patients while 44 (33.33%) patients were having constipation and 11 (16%) patients had GIB (Table 3).

**Table 3:** Precipitating factors of study patients with hepatic encephalopathy

Precipitating factors	Group A (n=44)	Group B (n=44)	Group C (n=44)
Spontaneous bacterial peritonitis, n (%)	7(15.9)	7(15.9)	10(22.7)
Respiratory tract infection, n (%)	6(13.6)	8(18.2)	5(11.4)
Urinary tract infection, n (%)	8(18.2)	7(15.9)	3(6.8)
Fever of unknown origin, n (%)	1(2.3)	1(2.3)	2(4.5)
Dyselectrolytemia, n (%)	20(45.5)	18(40.9)	16(36.3)
Constipation, n (%)	12(27.3)	14(31.8)	18(40.9)
Gastrointestinal bleeding, n(%)	9(20.5)	6(13.6)	6(13.6)

Evaluation of outcome

**Figure 2:**
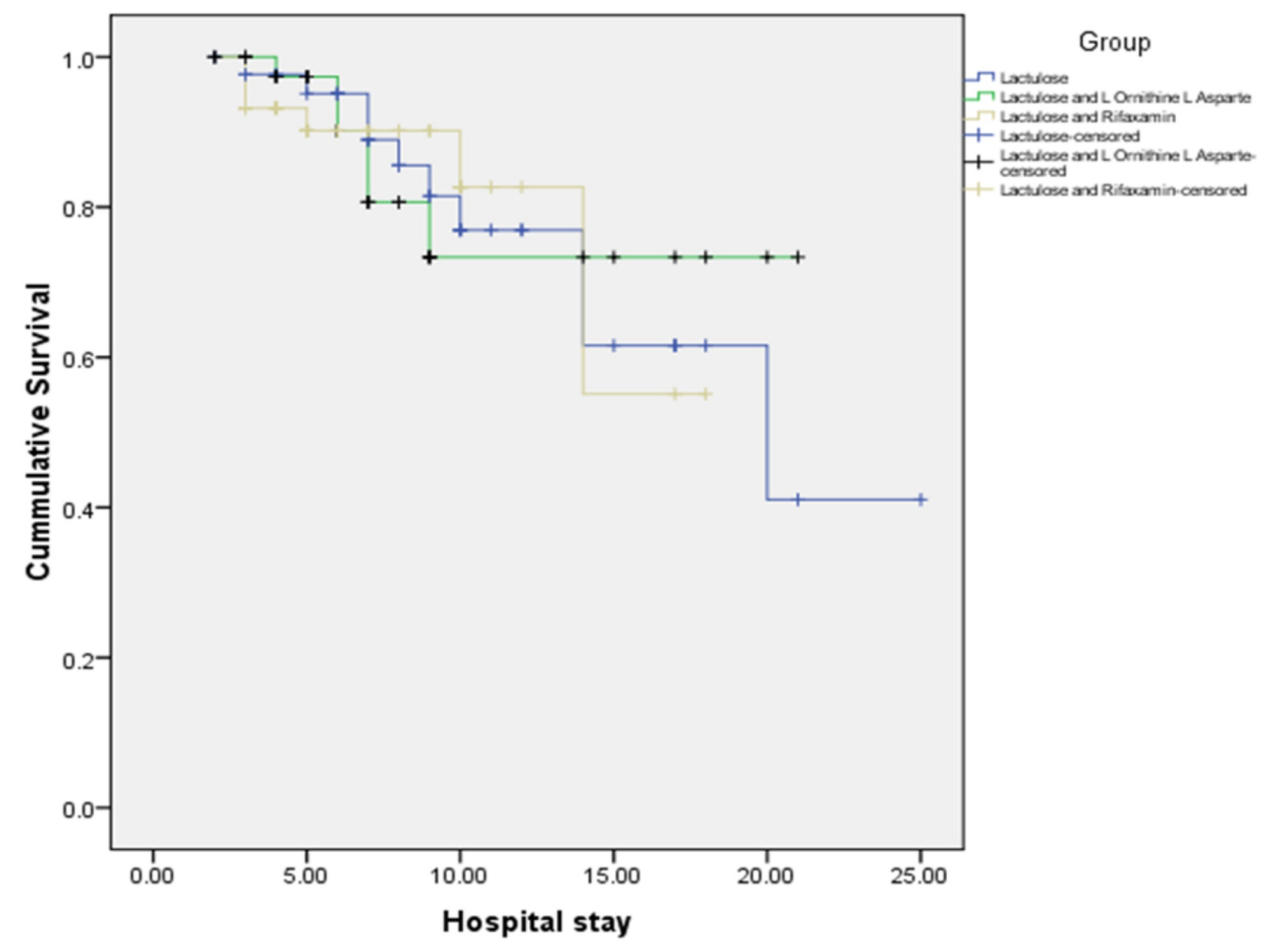
Kaplan–Meier survival curve for the three treatment groups during the hospital stay

The mean hospital stay in the study population was 9.64 ± 5.28, 7.36 ± 4.58, and 7 ± 3.69 in group A, group B, and group C, respectively (p=0.015). So patients in the lactulose plus LOLA and lactulose plus rifaximin groups had a shorter hospital stay as compared with the lactulose-alone group. Kaplan-Meier survival (Figure 2) for the three treatment groups showed that survival during the hospital stay was highest for the group, which received lactulose and LOLA, followed by the group with lactulose plus rifaximin. Survival was lowest for the group that received lactulose only.

Treatment response

In group A, 27 (61.4%) patients fully recovered from HE with lactulose only, in group B, 37 (84.1%) patients fully recovered from HE with lactulose and LOLA, and in group C too, 37 (84.1%) patients had a complete reversal of HE. This difference was statistically significant (p=0.017). There was a decrease in mortality in the lactulose plus rifaximin group and lactulose plus LOLA group with six (13.6 %) each in groups A and B vs. lactulose alone 10 (22.7%) with p-value <0.269. Out of 132 patients enrolled in our study, mortality was seen in 22 (16.7%) patients, and it was found that the mortality rate was signiﬁcantly higher in child class C patients.

Predictors of non-response to therapy in all three groups had a higher MELD score (30.59±7.25 vs. 20.54±6.41), higher Child score (12.32±1.29 vs. 9.83±1.74), and baseline higher total leukocyte count (17482±9668.6 vs 10375±8529.14) as compared to the patients who responded (p<0.001).

## Discussion

The identification and treatment of the precipitating factor is the most important aspect of the management of acute HE since symptoms of overt HE are debilitating and lead to non-adherence to a therapeutic regimen, which, in turn, leads to severe symptoms, frequent hospitalizations, and poor quality of life [4].

There had been some differences in the common precipitation factors across the studies. SBP was the most common precipitating factor reported in our study. This was consistent with a study done in Pakistan by Mumtaz et al. and Abid et al. who reported SBP as the most common precipitant in HE [5-6]. Our finding is, however, contradicting studies done in the USA by Souheil et al., who observed that infections were responsible in only 3% of cases. This could be related to adherence to therapy and regular monitoring of patients with LC in the USA, which resulted in early detection and treatment of infections [7].

In our study, we observed electrolyte imbalance to be present in 41% patients, and among electrolyte disorders, hyponatremia was much more common than hypokalemia in our patients, which was in agreement with other studies, such as Abid et al. and Alam et al. [6-8].

Constipation was seen in 33.7% in our study, which was consistent with the study by Abid et al. who reported that 21.7% of patients had constipation. However, our finding contradicts the study done by Zakaria et al., who reported constipation as a precipitating factor in only 7% of patients. Gastrointestinal bleeding (GIB) was identified only in 16% in our study, which is similar to the study done by Souheil et al., who also reported GIB in 18% of cases [7,9]. This was contrary to the findings of Bustamante et al. and Mehboob et al., who reported that GIB was the second and third commonest precipitating factor, respectively [10-11]. This can be attributed to a larger number of patients with large varices, which carry the risk of variceal bleed. The intake of a large amount of protein diet, especially meat and meat products, was also found in 16% patients as a precipitant factor in our study, which is similar to the study done by Devrajani et al. Similar to Mumtaz et al.'s study, the majority of our patients were grade II HE [5,12].

Lactulose, a non-absorbable disaccharide inhibiting intestinal ammonia production by lowering the colonic pH, is currently recommended as the first-line pharmacological treatment for HE [13]. In our study, only 61.4% of patients fully recovered from HE with lactulose alone and 22.7% had mortality. LOLA, a stable salt of the natural LOLA and aspartic acid, has been shown to improve HE [14]. Abdo-Francis et al. observed that treatment with LOLA was more effective than lactulose in improving HE, which is similar to our study [15]. However, in our study, we used LOLA plus lactulose in group B in which 84.1% of patients reverted back to normal. Only 2.3% of patients had treatment failure and 13.6% of patients died in the lactulose plus LOLA group. To our knowledge, there are no other studies comparing the combination of LOLA and lactulose with lactulose alone. Therefore, more studies are required to derive optimal results. However, LOLA infusion appears to be a safe and effective treatment of HE in our study.

Our study showed the superiority of rifaximin plus lactulose therapy over treatment with lactulose alone. We found that recovery from HE was 84% in group C, which is similar to the study done by Sharma et al., which showed that the combination of lactulose plus rifaximin is more effective than lactulose alone in the treatment of overt HE [16]. The risk of bacterial resistance appears to be lower with rifaximin than with systemic antibiotics. The plasma levels of rifaximin are negligible; therefore, bacteria outside the gastrointestinal tract are not exposed to appreciable selective pressure [17]. Thus, Rifaximin may be used in the long term or can be used several times during overt HE.

Our study is also supported by Bass et al., who also showed the superiority of rifaximin therapy over treatment with lactulose alone. More than 90% of patients received concomitant lactulose during the study period, and a significant treatment effect was noted [18]. Regardless of the decrease in mortality in the lactulose plus rifaximin group and the lactulose plus LOLA group in our study, it was not significant. However, Sharma et al. observed a significant decrease in mortality after treatment with lactulose plus rifaximin vs. lactulose and placebo [16].

The limitations of our study include it involving a single center and the small sample size. Further, large-scale, multicenter trials should be evaluated using robust clinical outcomes.

## Conclusions

Infection, especially SBP, is the commonest precipitating factors of HE followed by electrolyte imbalance, constipation, and GIB. The combination of lactulose either with LOLA or rifaximin is equally more effective in improving HE and reducing the duration of hospital stay than lactulose alone.
